# Supplementary material for: VEGF signaling regulates the fate of obstructed capillaries in mouse cortex
Source: eLife. 2018 Apr 26;7:e33670. doi: 10.7554/eLife.33670 (PMC5919759; doi:10.7554/eLife.33670)
Supplement: Supplementary file 2. [file elife-33670-supp2.pdf]

Contents

- Capillairy Loss Modeling based on experiemntal data
- Import data from spreadsheet
- Import the data
- CD to open excel files with branch order and risk distrubutions
- File is named mod_data
- Clear temporary variables
- moddata is excel sheet with obstruction risk for each branch order (min 1, max 16 depending on how risk is split) in order

Capillairy Loss Modeling based on experiemntal data

Written by Patrick Reeson Brown Lab, Division of Medical Sciences University of Victoria, Victoria BC Canada

```
% capillary branch order distrubution and obstruction risk loaded from
% excel file
% risk of pruning is 0.3
```

Import data from spreadsheet

Script for importing data from the following spreadsheet:

```
Workbook: C:\Users\P.Reeson\Documents\MATLAB\mod_data.xlsx
Worksheet: Sheet1
```

To extend the code for use with different selected data or a different spreadsheet, generate a function instead of a script.

```
% Auto-generated by MATLAB on 2017/07/06 17:30:37
% clear variables
clear
```

Import the data

CD to open excel files with branch order and risk distrubutions

File is named mod_data

mod_data (risk distrubuted across all branch orders based on obstruction distrubution)

```
moddata = xlsread('C:\Users\P.Reeson\Documents\MATLAB\mod_data.xlsx','Sheet1');
```

Clear temporary variables

```
clearvars raw;
```

moddata is excel sheet with obstruction risk for each branch order (min 1, max 16 depending on how risk is split) in order

```
moddata;

% List of all Branch Orders considered
brancho = moddata(:,1);
% get number of Branch Order Bins
nbin = brancho(end);
% Frequency of each Branching Order bin (based on real in vivo data, either
% for each bin or pooled if risk was also pooled (based on moddata file)
binfrq = moddata(:,2);
% Risk of Obstruction for each bin based on pooling stratagey, sum always
% adds up to the experiemntally observed risk for all vessels
obstrisk = moddata(:,3);
% Starting number of vessels for theoretical 100k cappilairies based on
% experimentally observed distrubution of branch orders and stratagey for
% pooling risk
startves = moddata(:,4);
% Prune risk is 30% of all obstructed
prunerisk = obstrisk .*0.30;
% Start matrix that will be the model, each row is a branch order's #
% vessels, eacg column is next iteration of 2 hour cycle of obstruction and
% pruning
vmodel = [brancho,startves];
% number of cycles to run
run = 50000;

% Run loop for each 2 hour window pruning iteration
i = 3;
k = 3;
% start with starting distrubution of vessels across branching orders
vmodel(:,1) = startves;
% newves is new # of vessels for each branch order after 1 2 hour window
newves = startves;
for i = 3:run-2
    iprune = newves .* prunerisk;
    newves = newves - iprune;
    vmodel(:,k) = newves;
        i = i+1;
        k = k+1;
end


% tworun is the total number of hours passed, ie number of 2 hours cycles
tworun = run*2;
xaxis = linspace(2,tworun,run-2);
vtime = transpose(xaxis);
% vsum is total sum of vessels at any time
vsum = sum(vmodel,1);
% vnorm is the normalized number of vessels for each branch order,
% normalized to vsum
vnorm = vsum./100000;
vnormt = vnorm.';
%tvsum is vsum transposed for graph
tvsum = vsum.';
```

```
vtime_2 = horzcat(vtime,tvsum);

% Generate each bin as fraction of total vessels for each time
q = 1;
z = 1;

for q = 1:run-2
    for z = 1:nbin
    binf(z,q) = vmodel(z,q)/vsum(q);
    z=z+1;
    end
    q=q+1;
end

figure
% Create xlabel
xlabel('Time (hours)');


set(0,'defaultlinelinewidth',2);
hold on


ax1 = subplot(1,3,1);
plot(ax1,vtime,vnormt)
ax2 = subplot(1,3,2);
p = 1;

ax3 = subplot(1,3,3);
r = 1;
for r = 1:nbin
    plot(ax3,vtime, binf(r,:))
    hold on
end
hold on

% select risk is from BO distrubutions based on gettin x number of
% obstructions OVERALL if only at select risk vessels obstructed

u = 1;
a = 1;
vsummat = vsum;
% Concat vert vsum to get a nbinXrun-2 matrix
for a = 1:nbin-1
    vsummat = vertcat(vsummat,vsum);
    a=a+1;
end


for u = 1:run-2
 % number of at risk vessels
        riskfinal = obstrisk.*vmodel(:,u);
        % normalized to  number of vessels in the branch order
        risknorm = riskfinal/vsummat(:,u);
        % sum of all  risk across branch orders
        sumriskfinal(:,u) = sum(risknorm,1);
```

```
    u = u+1;
end
% starting numbers of at risk population, ie zero is time zero
zerorisk = sumriskfinal(1,1);
% Normalized sumrisk final
nsrf = sumriskfinal./ zerorisk;
 ax4 = subplot(1,3,2);
 plot(ax4, vtime, nsrf(1,:));

set(ax1,'FontName','Calibri','FontSize',14,'FontWeight','bold',...
    'LineWidth',2,'XColor',[0 0 0],'YColor',[0 0 0],'ZColor',[0 0 0]);
% Set the remaining axes properties
set(ax3,'FontName','Calibri','FontSize',14,'FontWeight','bold',...
    'LineWidth',2,'XColor',[0 0 0],'YColor',[0 0 0],'ZColor',[0 0 0]);

 set(ax4,'FontName','Calibri','FontSize',14,'FontWeight','bold',...
    'LineWidth',2,'XColor',[0 0 0],'YColor',[0 0 0],'ZColor',[0 0 0]);
% Create legend
legend1 = legend(ax4,'show');
set(legend1,...
    'Position',[0.906051734112396 0.140358770176528 0.0739385045595783 0.795792056634875]);
title(legend1,'Branch order');
```

Published with MATLAB® R2017a
